# The nuclear pore complex as a spatial organizing hub for high-risk DNA lesions

**DOI:** 10.1080/19491034.2026.2709897

**Published:** 2026-07-30

**Authors:** Dante DeAscanis, Boeun Nam, Hongseon Song, Younghoon Kee

**Affiliations:** a Department of Molecular Biosciences, College of Arts and Sciences, University of South Florida, Tampa, FL, USA; b Department of New Biology, Daegu Gyeongbuk Institute of Science and Technology (DGIST), Daegu, Republic of Korea

**Keywords:** DNA repair, double strand break, nuclear pore complex, replication fork, transcription control

## Abstract

The nuclear pore complex (NPC) is best known for nucleocytoplasmic transport, but growing evidence implicates nucleoporins and NPC-associated environments in genome maintenance. Here, we discuss the NPC as a spatial organizer of hazardous DNA lesions rather than a passive transport channel. Three functions recur across systems. First, NPC-associated environments can quarantine lesions arising in repetitive DNA, heterochromatin, dysfunctional telomeres, and other high-risk chromatin contexts. Second, nuclear pore association can influence recombination and repair pathway choice. Third, mobile off-pore nucleoporins may act directly at damaged chromatin to stabilize repair-associated chromatin states and restrain harmful RNA-containing intermediates. We distinguish direct mechanistic insight from evidence based on spatial association or genetic perturbation and highlight unresolved questions about NPC heterogeneity, lesion mobility, and protective versus mutagenic repair. This view positions nucleoporins as conditional regulators of genome stability.

## Introduction

**Figure 1.**
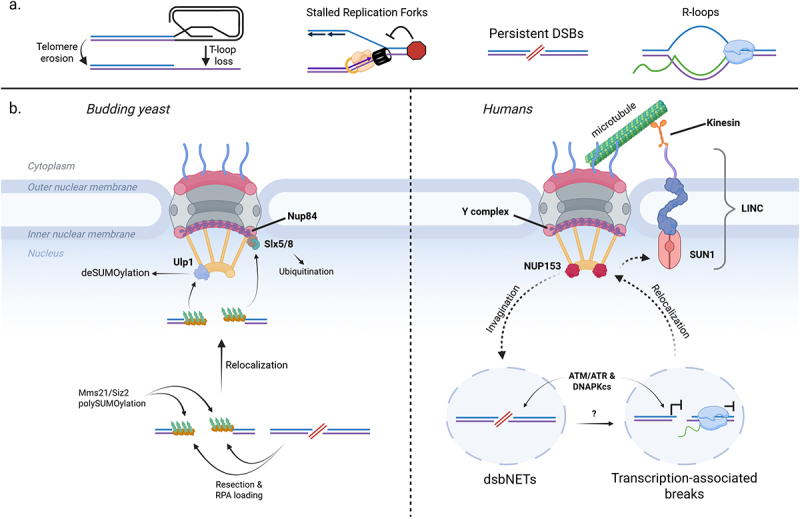
Conserved logic and divergent execution of nucleoporin-mediated lesion organization across species. High-risk DNA lesions are spatially reorganized away from chromatin environments that are unfavorable for safe repair. a. Eroded telomeres, stalled replication forks, persistent DSBs, and R-loops are high-risk lesions that interact with the NPC to promote context-dependent repair mechanisms. b. *(left)* in budding yeast, persistent DSBs, stalled forks, and eroded telomeres engage relatively well-defined NPC-linked SUMO circuitry involving the Nup84 complex, Slx5/8, and Ulp1. After end resection and RPA loading, polySumoylation signals recruit Slx5/8 which mobilizes the break to the nuclear periphery, anchoring the lesion through interactions with Nup84. Ubiquitin chains are either resolved through Ulp1-mediated deSumoylation or proteasomal degradation, leading to specific repair outcomes. *(right)* in humans, analogous lesion handling appears more heterogeneous and may involve mobilization, LINC-envelope tethering, dsbNET-like membrane invaginations, and lesion-associated nucleoplasmic nucleoporin assemblies. Dashed connections denote mechanisms that remain less firmly established. Created using BioRender.

Genome integrity depends not only on lesion recognition and repair biochemistry, but also on where damaged DNA is processed within the nucleus. This spatial dimension becomes especially important when lesions arise within repetitive DNA, transcriptionally active chromatin, telomeres, or stressed replication forks (Figure 1a). Here, repair is intrinsically hazardous and can generate ectopic recombination events or chromosomal rearrangements.

The nuclear pore complex (NPC) is a large multi-subunit assembly embedded in the nuclear envelope and classically viewed as the gateway for nucleocytoplasmic transport [1,2]. In most eukaryotes, the NPC is assembled from ~30 nucleoporins (Nups) organized into cytoplasmic and nucleoplasmic rings, an inner ring, membrane-anchoring elements, and a central phenylalanine-glycine (FG)-repeat network that mediates selective transport [3,4]. Beyond this canonical role, many studies now connect nucleoporins to genome organization [5], transcriptional control [6,7], DNA repair [8–10], and replication stress responses [11–13].

These observations have often been discussed under the broad idea that the NPC contributes to genome maintenance through both transport-dependent and transport-independent mechanisms [14–16]. However, the transport-independent arm of this literature remains conceptually diffuse. Here we focus on a specific question: how do NPC-associated environments and off-pore nucleoporin pools help cells process lesions that are especially prone to aberrant repair?

We argue that the available literature converges on three recurring functions. First, NPC-associated environments can quarantine high-risk lesions by relocating them away from hazardous chromatin neighborhoods [9,17–20]. Additionally, Chiolo et al. highlighted how NPC-mediated lesion mobility cooperates with genome architecture to stabilize DNA repair, further supporting lesion sequestration as a driver of repair fidelity [21]. Second, pore-proximal environments can shape repair outcomes by controlling when and how recombination proceeds [8,12,22]. Third, soluble off-pore nucleoporins can restrain damage locally in transcriptionally distinct loci [15,16,23]. Viewed from this perspective, the NPC is not merely a structural landmark at the nuclear periphery, but a spatial organizer that helps determine where lesions are handled and which forms of repair become permissible (Table 1).

**Table 1.** Nucleoporins in DNA repair across species.

Species	DNA Lesion	Nups	Interactors	Function	Repair Pathway	References
Budding yeast	DSBs	Nup84	Ulp1^1^	Relocalization requires SUMOylation signal	–	Palancade et al.[10]^1^, Nagai et al. [9]
Budding yeast	DSBs	Nup84	Siz2, Mms1	PolySUMOylation deposited via Siz2/Mms1 directing to NPC to promote alternative repair pathways	MMEJ, BIR	Horigome et al. [8]
Budding yeast	DSBs	Nup84	Cik1-Kar3	The Cik1-Kar3 kinesin-14 complex promotes DNA mobility of subtelomeric DSBs and tethering to Nup84	BIR	Chung et al. [33]
Budding yeast	Stalled Replication Forks	Nup84	Slx5/8, Smc5/6, RPA^2^ Slx5/8 interacts with Nup84 and CAG repeat-induced stalled forks to promote Rad51-mediated fork restart		–	Su et al. [13], Whalen et al. [44]^2^
Budding yeast	Stalled Replication Forks	Nup84^3^, Nup1^4^	–	Nup1 C-terminal mutants prevent movement of stalled forks at CAG repeats and telomeres to the NPC promoting error-prone recombination	Unequal SCR	Gaillard et al. [37]^3^, Aguilera et al. [55]^4^
Budding yeast	Stalled Replication Forks	Nup84^5^, Nup132^5,6^, Nup133^5,6^	Pli1^5^, Slx5/8^5^, Ulp1^6^ PolySUMOylation via Pli1 prevents RAD51 strand invasion prior to anchorage to the NPC where Ulp1 de-conjugates SUMO to promote repair		RDR	Kramarz et al. [22]^5^, Schirmeisen et al. [12]^6^
Budding yeast	R-loops	Nup170, Nup157, Nup60	RPA, Mms21	ssDNA coated by Mms21-dependent monoSUMOylated RPA promotes sensing of R-loops to the NPC to reduce R-loop toxicity burden	–	Penzo et al. [60]
Budding yeast	Eroded Telomeres	Nup84	Slx5/8, RPA	ssDNA ends are coated with RPA and SUMOylated forming “congested” intermediates that are signaling to the NPC in a Slx5/8 dependent-manner to promote subsequent repair attempts	Type II Recombination	Churikov et al. [32]
Fruit flies	DSBs	Nup98	SMC5/6, HP1	Nup98 is recruited to repair sites forming immiscible conden-sates with HP1 and mobilize repair sites, excluding RAD51	HR	Merigliano et al. [15]
Humans	DSBs	NUP153, NUP50	53BP1	NUP153 is required for 53BP1 import and intranuclear targeting to DSBs with NUP50 promoting an antagonistic relationship between 53BP1 and BRCA1	NHEJ	Moudry et al. [14], Mackay et al. [30]
Humans	DSBs	NUP153	LINC, KIF5B, KIF13 The NE invaginates via KIF5B/KIF13 and LINC coordination to expose DSBs to the NPC promoting faithful repair		NHEJ	Shokrollahi et al. [31]
Humans	DSBs	NUP153	SUN1, PER2	PER2 targets DSBs to the NPC where it is docked via NUP153 and SUN1 promoting RAD51 assembly and repair	HR	Bozec et al. [28]
Humans	DSBs	NUP107 complex	ATM, PRC1	Intranuclear NUP107 and NUP43 colocalize to DSBs interdependent with PRC1 members to promote transcriptional repression at DSBs in an ATM-dependent manner	HR	Song et al. [16]
Humans	R-loops	Tpr	GANP	Tpr cooperates with GANP to resolve RNA:DNA intermediates where depletion of Tpr results in increased replication stress and genomic instability	–	Kosar et al. [61]
Humans	Telomere maintenance	NUP153, NUP62, Tpr	POT1	In POT1 mutated cells, the NPC anchors and promotes mitotic DNA synthesis of the dysfunctional telomeres	–	Pinzaru et al. [66]

Notes: Superscripts indicate the specific reference supporting the associated protein interaction or mechanistic detail when multiple studies are cited within a single table entry. A dash (–) in the Repair Pathway column indicates that no specific repair pathway has been elucidated or reported.

*Abbreviations*: BIR, break-induced replication; DSB, double-strand break; HR, homologous recombination; MMEJ, microhomology-mediated end joining; NE, nuclear envelope; NHEJ, non-homologous end joining; NPC, nuclear pore complex; RDR, recombination-dependent replication; SCR, sister chromatid recombination.

## Terminology and limits of current evidence

Because this literature spans *S. cerevisiae* (budding yeast), *D. melanogaster* (fruit flies), and mammals, terminology requires care. In this review, NPC refers to the assembled pore complex at the nuclear envelope. Nucleoporins (or Nups) refer to the individual protein constituents of the NPC. We use NPC-associated to describe events occurring at or immediately adjacent to intact pores or closely related nuclear-envelope environments. We use ‘off-pore’ to describe mobile nucleoporin pools that function within the nucleoplasm independently of assembled pores.

We also distinguish between transport-dependent and transport-independent interpretations, but with caution. In some studies, depletion of a nucleoporin can alter nuclear transport [14,24], RNA metabolism [25], transcription [7], cell-cycle progression [23], or chromatin state [26,27], making it difficult to conclude that an observed repair phenotype reflects a direct local role at the lesion. This issue is especially important for multifunctional nucleoporins such as NUP153 [14,28–31].

A second caution concerns physical linkage. Much of the field relies on chromatin immunoprecipitation, proximity-based nascent DNA proteomics, or microscopy colocalization. These approaches can strongly support lesion association, relocalization, or local enrichment, but they do not by themselves establish direct physical tethering between a nucleoporin and damaged DNA. Accordingly, we preferentially use terms such as association, proximity, relocalization, and NPC-linked unless direct physical interaction has been demonstrated. These distinctions are central to interpreting the field. They help separate what is firmly established from what is genetically implicated, proximity-based, or still mechanistically unresolved.

## Spatial quarantine of high-risk DNA lesions

Not all DNA lesions carry the same repair-associated risk. Persistent double-strand breaks (DSBs) in repetitive DNA, heterochromatin, sub-telomeric regions, or eroded telomeres-summarized in (Table 1)-increase the likelihood of ectopic recombination and chromosomal rearrangements and are therefore preferentially resolved through SUMO-dependent repair [8,13,15,32]. Additionally, these risks are shaped by SUMO-dependent damage processing and by the chromatin state of subtelomeric domains [33,34]. A recurring solution across systems is to move such lesions away from their original chromatin context and process them in a distinct perinuclear environment (Figure 1(b), left).

This principle has been most clearly defined in budding yeast, particularly in strains lacking the homologous donor sequence for repair of the MAT locus. Upon DSB induction via the HO-endonuclease targeting the *MAT* locus, persistent or difficult-to-repair DSBs relocalize to the nuclear periphery through SUMO-dependent pathways [8,9,35]. Siz2 and Mms21 mediate the polySUMOylation of various target proteins, thereby promoting SUMO-dependent targeting to the nuclear-pore. This damage-induced SUMOylation is facilitated by RPA-coated ssDNA through activation of Siz2 [8,33]. The subsequent polySUMOylation signal triggers recruitment of the SUMO-targeted ubiquitin ligase (STUbL) Slx5/Slx8, which is required for movement to the periphery and subsequent docking to the Nup84 complex at the nuclear pore [8,9]. Here, ubiquitinated targets are signaled for proteasomal degradation, triggering break-induced replication (BIR) as a favored mechanism, despite being error-prone [8,9]. Additionally, Ulp1, a SUMO-dependent protease that is positioned at the NPC and stabilized by Nup84, facilitates deSUMOylation and promotes DNA repair in a non-redundant manner [10,12]. Concomitantly, sub-telomeric and repeat-associated lesions similarly depend on NPC-linked relocation, and failure of this routing correlates with aberrant recombination outcomes [13,36,37]. In this context, the nuclear periphery appears to serve not simply as a docking site, but as a repair compartment that spatially separates hazardous intermediates.

A similar logic is evident in metazoans, although the participating architecture is more diverse. In fruit flies, heterochromatic DSBs move out of the HP1a-rich domain and toward the nuclear periphery before homologous recombination proceeds. Recent work has highlighted a role for off-pore Nup98 condensates in mobilizing heterochromatic breaks and excluding Rad51 at an early stage [15,18,20]. Nup98 is an FG-repeat containing nucleoporin capable of forming liquid-liquid phase separation (LLPS) condensates [15,38,39], and upon its recruitment to heterochromatin breaks in the HP1 domain, immiscible condensates are formed around the lesions [15]. The phase-separated condensates are then pushed to the surface via capillary forces, and from there, nuclear F-actin filaments cooperate with myosins to ‘walk’ the condensates to the NPC [15,17,20]. Once at the NPC, it is docked through interactions with the Nup107-160 (Y-complex) [20,40]. In mammalian systems, NUP153, SUN1, and LINC components cooperate with kinesin-driven microtubules to generate DSB-capturing nuclear envelope tubules (dsbNETs) capable of invaginating the nuclear envelope to sequester breaks [31]. When considering transcriptional dynamics, transcription-coupled DSBs (TC-DSBs) have been shown to relocate to the nuclear envelope in a PER2-dependent manner. PER2 and the circadian clock regulate TC-DSB targeting to the nuclear envelope through a pathway involving NUP153, SUN1, and LINC-associated nuclear-envelope components [28]. However, SUN1 recruitment to TC-DSBs was reported to be independent of DNA damage response kinases ATM, ATR, or DNA-PKcs while these kinases were required for dsbNET-mediated invagination [28,31]. It is possible that these mechanisms cooperate, given their shared reliance on NUP153, SUN1, and LINC components, but this remains unresolved [28,30,31]. (Figure 1b, right)

Nonetheless, the aggregate of these observations suggests that NPC-mediated lesion sequestration functions primarily to mitigate aberrant repair. Importantly, the unifying feature is not necessarily the tethering machinery or target architecture, but the ‘need’ to remove high-risk lesions from chromatin environments that favor inappropriate interactions. In this view, NPC-associated environments function as spatial quarantine zones that reduce the chance of illegitimate recombination by separating hazardous lesions from competing templates and nearby breaks.

This model also helps explain the consequences of failed perinuclear capture. For instance, in mammalian cells, disruption of dsbNETs can promote lesion clustering within the nucleoplasm [31]. Additionally, disrupting TC-DSBs mobility to the nuclear periphery primes the formation of DNA damage-rich compartments, coined D-compartments, that may concentrate repair factors locally but also increase the danger of trans interactions between distinct breaks in adjacent loci [28,31,41,42]. Thus, the primary benefit of peripheral routing may be the physical segregation of lesions from their surroundings.

## NPC-associated environments shape repair pathway choice

Spatial quarantine alone may not explain the ultimate repair outcome. Once a lesion reaches an NPC-associated environment, cells may still determine when recombination becomes permissible and which repair pathway is used. A strong mechanistic insight again comes from budding yeast, where lesion relocalization and repair outcome are linked through SUMO-dependent circuitry. SUMO modifications can contribute both to lesion relocalization to the NPC and to determining how lesions are ultimately repaired [9,10,12,22,32]. PolySUMOylation can promote lesion relocalization to NPCs through Slx5/8-dependent pathways, whereas de-SUMOylation at NPC-associated sites may further influence whether repair proceeds through conservative homologous recombination or more mutagenic pathways such BIR or microhomology-mediated end joining (MMEJ) [8,9,22,32,43]. Arrested forks and eroded telomeres illustrate this logic particularly well. In fission yeast, DSB-free arrested forks relocalize to NPC-associated environments after Rad51 loading and activity; Pli1-dependent polySUMOylation promotes NPC anchorage but restrains HR-mediated DNA synthesis until NPC-associated Ulp1 and proteasome activities remove SUMO conjugates and permit recombination-dependent replication. In budding yeast, eroded telomeres similarly relocalize to NPCs through SUMO – STUbL-dependent pathways, where NPC association promotes recombination-mediated telomere elongation via type II recombination [22,32]. Furthermore, de-SUMOylation by Ulp1 allows for the initiation of restarted DNA synthesis once bound to the NPC, thereby preventing premature or ectopic Rad51-dependent replication [12,13,22,44]. At telomeres, critically short or dysfunctional telomeres also engage in NPC-linked SUMO pathways, promoting type II recombination-dependent survival programs that can preserve viability but at the cost of increased genomic instability [32,45]. These examples indicate that the nuclear periphery can act both as a protective environment and as a compartment that permits last-resort repair strategies when lesions persist.

In mammals, the link between NPC association and repair outcome is less clearly defined. Studies in human cells support lesion anchoring, chromatin regulation, and local control of repair factor assembly [28,31,41,46–48], but clear counterparts of the yeast NPC-linked BIR-like survival program have not yet been established [8]. For that reason, it would be premature to assign a single repair outcome to mammalian NPC-associated environments. A more cautious view is that these environments can influence both lesion isolation and recombination competence, while the final outcome likely depends on lesion persistence and chromatin context.

## Off-pore nucleoporins as local chromatin and transcriptional regulators

A key development in the field has been the recognition that not all nucleoporin-dependent genome maintenance occurs at the nuclear envelope. Several nucleoporins, including NUP98 and NUP153, can occupy nucleoplasmic chromatin and participate in transcriptional regulation, enhancer function, and higher-order genome organization [23,29,49–51]. These chromatin-associated functions make it plausible that mobile nucleoporin pools are redeployed during DNA damage to remodel local lesion environments. Off-pore nucleoporins may influence damage responses in at least two ways: by forming condensate-like environments that restrict repair-factor access, and by associating with damaged chromatin to reinforce transcriptional repression or chromatin stabilization.

**Figure 2.**
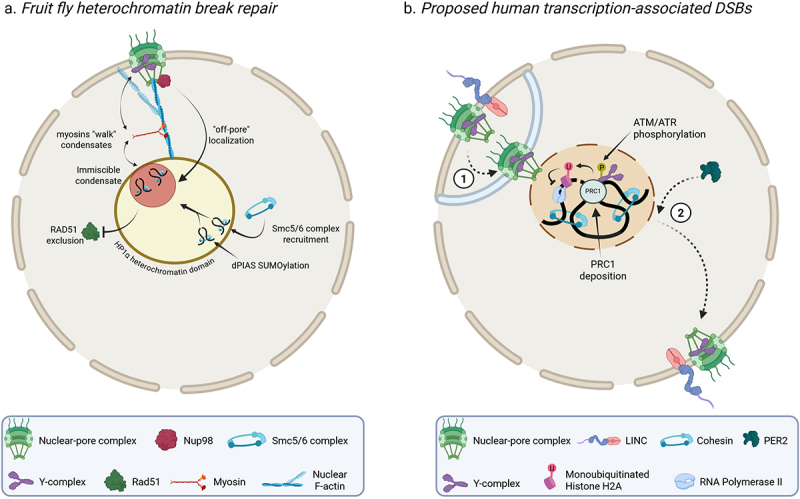
Context-dependent nucleoporin functions beyond canonical NPC tethering. a. Current working model for an off-pore nucleoporin-mediated heterochromatin break repair in *Drosophila*. Damage occurring in the HP1α domain is particularly prone to ectopic recombination with adjacent repeat regions. The Smc5/6 complex is recruited to the DSB where it is SUMOylated via the SUMO E3-ligase dPIAS. This SUMOylation signal then triggers the recruitment of Nup98 to DSBs, forming an immiscible condensate within the HP1α domain. The phase-separated condensate inhibits Rad51 accessibility to the lesion; capillary forces push the Nup98 condensate to the surface of the HP1α domain. Thereafter, nuclear F-actin filaments and myosins cooperate to ‘walk’ the condensate to the nuclear periphery where it is anchored by the Nup107-containing Y-complex. b. A proposed model for processing transcription-associated DSBs in humans. Before damage, actively transcribed chromatin is associated with elongating RNA polymerase II. After DSB induction, ATM is recruited to damaged chromatin. Nucleoplasmic Y-complex components, including NUP107 and NUP43, are proposed to be a phosphorylated substrate of ATM while simultaneously physically associating with PRC1 factors *in cis* to damage chromatin. PRC1 subsequently monoubiquitinates histone H2A, and in this model, nucleoporins and polycomb proteins promote localized transcriptional repression, stabilize damaged chromatin, and limit harmful RNA-containing intermediates during repair. Independent of the localized transcription-associated break repair, 1) dsbNETs may invaginate to capture the lesions to promote safe repair by blocking access to adjacent active loci, or 2) the damaged chromatin may tether to the nuclear envelope via cooperations between SUN1 and the NPC in a PER2 dependent-manner. Dashed arrows indicate speculative or incompletely defined steps. Created using BioRender.

The clearest metazoan example comes from *Drosophila* heterochromatin breaks, where off-pore Nup98 contributes to early break handling together with HP1- and SMC5/6-enriched environments (Figure 2a), helping mobilize lesions and restrain Rad51 before repair proceeds in a safer location [15,17,20]. Here, nucleoporins are not simply passive markers of a destination at the periphery; they appear to help establish repair-permissive chromatin states.

A related idea is emerging in mammalian TC-DSB responses. When DSBs occur in actively transcribed chromatin, cells face a dual challenge: they must repair the break while preventing continued transcription, polymerase collisions, and the accumulation of harmful RNA-containing intermediates [41,47,52]. Transcriptional activity can favor homologous recombination in some settings, yet it can also exacerbate conflict between ongoing RNA synthesis and repair [47,52,53]. Recent work suggested that nucleoplasmic pools of Y-complex components, including NUP107, NUP133, and NUP43, act together with Polycomb-repressive complex 1 (PRC1) factors in localized transcriptional repression and chromatin stabilization [16]. In this study, DSB-associated NUP signals were clearly separated from the nuclear periphery, consistent with a possible NPC-independent role for nucleoporins. Taken together, these observations support a working model in which off-pore nucleoporins help create a repair-permissive chromatin state at transcription-linked breaks rather than serving as structural tethers to assembled pores (Figure 2b). However, further investigations are needed to determine whether dsbNET-mediated invaginations, PER2-dependent TC-DSB tethering, and transcriptional repression are mechanistically connected. One unresolved question is how NPC- or nucleoporin-mediated transcriptional repression relates to DNA lesion clustering. Cohesin and CTCF are established regulators of damage-induced chromatin reorganization, and cohesin has been implicated in DSB-induced transcriptional repression [48]. In parallel, FG-containing nucleoporins such as NUP98 and NUP153 contain intrinsically disordered regions that can support condensate-like behavior and interact with chromatin-organizing factors, including CTCF/cohesin or BRD4 in non-damage contexts [5,29,51,54]. These observations raise the possibility that off-pore FG-NUPs help shape local repair compartments by selectively controlling repair-factor access while limiting transcriptional machinery near damaged chromatin. Whether such condensate-like NUP environments communicate with cohesin/CTCF-dependent DSB clustering or with nuclear-envelope targeting pathways such as dsbNET formation and Y-complex-associated relocation remains an important open question (Figure 2b).

## NPC-linked control of replication stress, R-loops, and RNA-associated lesions

Although replication stress is often discussed separately from DSB relocalization, both involve the spatial handling of unstable DNA intermediates. Stalled or collapsed replication forks generate unstable DNA structures that are highly vulnerable to unscheduled recombination, especially in repetitive DNA or transcribed regions [11,44,55,56]. In budding yeast, forks stalled or collapsed at triplet repeats can relocalize to NPC-associated environments, where restart is coordinated with SUMO-dependent signaling and controlled Rad51-dependent recombination [13,44]. A related SUMO-dependent NPC route operates at arrested forks in fission yeast, where post-anchoring SUMO removal by Ulp1 and proteasome activities permits recombination-dependent DNA synthesis [22]. A review by Boer et al. also highlighted each context-dependent repair strategy engaged at the NPC after lesion relocalization, emphasizing how distinct replication fork barriers dictate repair choice [57]. Together, these findings fit well with the broader model outlined above: pore association appears to help prevent premature strand invasion while permitting controlled recombination-dependent restart when simple fork recovery is no longer possible.

In mammalian cells, nascent-DNA proteomics have strengthened the case that nucleoporins are present near stressed replisomes. iPOND- and related proximity-based approaches detected enrichment of multiple nucleoporins, including TPR, NUP153, NUP107, and NUP133, at aphidicolin-stalled forks or in fork-proximal fractions [58,59]. These data support lesion proximity, but not necessarily direct fork engagement. Importantly, many perturbation studies in this area rely on depletion of nucleoporins that also affect transport, RNA processing, or transcription, so fork phenotypes must be interpreted cautiously.

This caution is especially important for RNA-associated lesions. In budding yeast, NPC-linked factors including Nup170, Nup157, and Nup60 have been genetically implicated in coping with R-loop-associated DNA breaks, and these responses are connected to SUMO signaling and perinuclear lesion handling [60]. In mammals, TPR and GANP have been implicated in limiting RNA-associated replication stress, but whether the observed phenotypes reflect direct control of RNA-DNA hybrid metabolism, local functions at damaged forks, or broader consequences of altered mRNA export remains unresolved [61]. This point is particularly relevant because TPR is functionally linked to TREX2-associated mRNA export, and phenotypes observed upon TPR depletion may therefore reflect both local genome-maintenance functions and indirect consequences of perturbed RNA export [62]. Two conserved RNA export machineries should also be distinguished here: the THO/TREX complex, which functions in transcription elongation [63], mRNA biogenesis [64], and suppression of co-transcriptional R-loops [65], and the TREX2 complex, which is physically associated with NPCs and contributes to gene gating and mRNA export [62].

Telomere crisis provides a useful connection between DSB repair and replication stress. In POT1-deficient human cells, dysfunctional telomeres relocalize to NPC-associated environments involving NUP153, NUP62, and TPR, where mitotic DNA synthesis contributes to cell survival [66]. As observed at eroded telomeres in budding yeast, the key point may be that the periphery supports salvage mechanisms for unusually difficult DNA intermediates. Taken together, stalled forks, RNA-linked lesions, and dysfunctional telomeres can be viewed as related versions of the same problem: how cells spatially manage repair-dangerous DNA intermediates.

## Future considerations

Despite substantial progress linking lesion relocalization to repair control, several key mechanistic questions must still be resolved before NPC-linked genome maintenance can be placed within a unified framework.

### Which structural interfaces anchor metazoan lesions?

Whereas the Nup84 complex provides a relatively well-defined NPC-linked platform in budding yeast, the relevant anchoring mechanisms in metazoans appear more heterogeneous. Current candidates include LINC-dependent tethering, PER2/SUN1-associated nuclear-envelope interactions, dsbNET-like membrane invaginations, and possibly lesion-associated nucleoplasmic pools of nucleoporins [15,20,28,31]. Recent work suggests that the intrinsically disordered N-terminal region of NUP107 may contribute to nucleoporin recruitment or retention at transcription-associated DSBs in mammalian cells [16]. In this setting, Polycomb factors involved in DSB-induced transcriptional silencing [67–70] may cooperate with nucleoporins to create local repressive environments that support spatial control of damaged chromatin. An important next step will be to distinguish stable anchoring mechanisms from transient capture events or indirect consequences of local chromatin remodeling. High-resolution interactomics and perturbation-based mapping should also clarify whether transcription-linked intermediates, including RNA-DNA hybrids, contribute to the recruitment of specific nucleoporins to damaged chromatin [16,52,53].

### Are all NPCs equivalent repair environments?

An emerging question is whether all pores are functionally interchangeable, or whether only molecularly distinct NPC subpopulations [71] engage damaged chromatin [12,34,72]. If repair-active pores differ in composition, associated basket proteins, or local signaling properties, this could help explain why only subsets of lesions appear to access NPC-linked pathways. To support this, evidence in budding yeast has proposed that the Ctf18-RFC is recruited to NPC subunits lacking the basket proteins Mlp1 and Mlp2 and directly interacts with Nup170 to mediate subtelomeric gene silencing [34]. While basketless NPC variants along with broader structural NPC variants have been highlighted in DNA repair (reviewed in Simon et al. [73]), defining whether repair-competent NPCs represent specialized microenvironments, rather than a uniform nuclear periphery, will be important for interpreting both lesion relocalization and repair outcome [15,28,31,73]. Indeed, recent evidence corroborates the notion of the inequivalence between distinct NPC-associations in facilitating repair. In fission yeast, Ulp1-associated NPCs promote stalled fork restart in a SUMO-dependent manner while proteasome-associated NPCs cooperate to promote fork progression. These two processes are mutually exclusive and cannot compensate for each other, indicating that distinct NPC environments are capable of promoting separate pathways [12].

### Do soluble nucleoporin subpopulations actively mediate repair?

Although the NPC is classically defined as a perinuclear structure, accumulating evidence suggests that mobile, off-pore nucleoporin pools may also participate directly in genome maintenance. Off-pore Nup98 assemblies in fruit flies provide one clear example, but the extent to which this principle applies to higher-order metazoans remains uncertain [15]. In mammals, a particularly important question is whether Y-complex components must remain physically linked to the nuclear envelope to influence DSB responses, or whether soluble nucleoplasmic pools can act locally through chromatin-regulatory partners such as PRC1 [16]. Resolving this issue will be essential for distinguishing lesion relocalization to the pore from local damage control mediated by mobile nucleoporins.

### Is there a conserved link between SUMO signaling, chromatin silencing, and early repair decisions?

SUMO-dependent restraint of premature recombination is a recurring theme in budding yeast and fruit fly systems, whereas localized transcriptional repression and chromatin compaction are emerging as important features of mammalian DSB control [8,9,15,16,43]. Whether these observations reflect a conserved regulatory logic remains unknown. In particular, it will be important to determine whether nucleoporins influence early repair decisions, including end resection, by coupling SUMO-dependent lesion handling to transient chromatin silencing and control of transcription-associated intermediates [16,47,52,53].

### Is the NPC-associated environment primarily protective, or can it also permit mutagenic repair?

**Figure 3.**
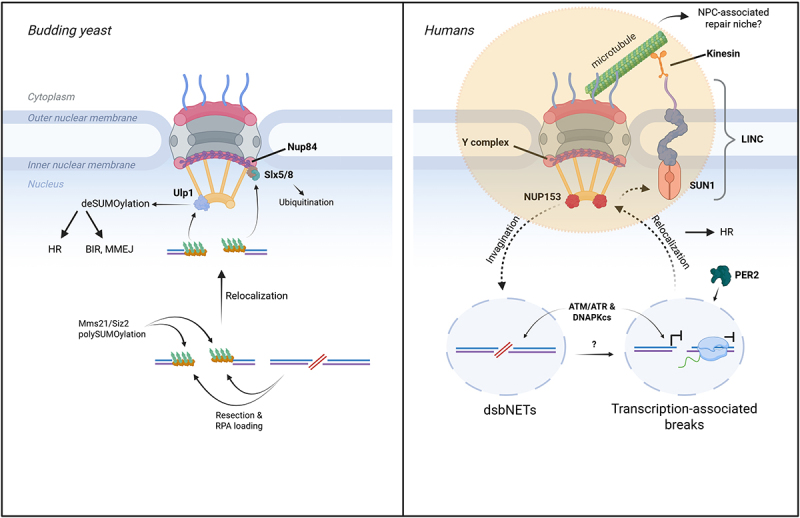
Protective versus mutagenic consequences of NPC-associated lesion compartmentalization. Conceptual model comparing the consequences of NPC-associated repair environments in budding yeast and humans. In budding yeast, persistent DNA lesions can be relocalized to NPC-associated repair sites, where repair pathway choice may diverge toward homologous recombination (HR) or alternative pathways such as break-induced replication (BIR) and microhomology-mediated end joining (MMEJ). By contrast, in human cells, nuclear-envelope-associated lesion capture and transcription-associated tethering have been linked to spatially controlled repair, but whether analogous NPC-associated repair niches directly influence pathway choice under conditions of persistent DNA damage remains unresolved. The figure highlights a central open question in the field: whether NPC-associated environments in metazoans function only to support protective repair or can also permit alternative, potentially mutagenic repair outcomes. Created using BioRender.

A central unresolved issue is whether NPC-associated repair environments act mainly to preserve genome integrity or, under the context of difficult-to-repair lesions, enable survival through more error-prone mechanisms. In mammalian systems, NPC-linked repair has often been interpreted as protective, for example by limiting inappropriate factor assembly, stabilizing damaged chromatin, or promoting repair in a more spatially controlled context [16,28,30]. By contrast, studies in budding yeast indicate that persistent lesions, including eroded telomeres and arrested forks, can engage NPC-linked SUMO circuitry that ultimately permits recombination-dependent DNA synthesis, including pathways related to BIR or microhomology-mediated end joining (MMEJ) [22,32,44]. Together, these observations suggest that NPC-associated repair environments may not exclusively function in a protective capacity but could also facilitate repair pathways with elevated mutagenic potential in the context of persistent DNA damage. Whether mammalian NPC-associated repair similarly influences repair pathway choice toward alternative, potentially mutagenic repair outcomes under persistent stress remains an important open question (Figure 3).

### Can transport-independent nucleoporin functions be exploited in cancer?

Nucleoporins are frequently dysregulated in cancers – influencing molecular trafficking [74–77], and in some settings, this may promote stress tolerance [78,79]. Solid tumors often upregulate specific nucleoporins [80–82], while rare and aggressive hematological malignancies, particularly acute myeloid leukemias (AMLs), harbor NUP98 fusion proteins [83–86]. These NUP98 oncofusion proteins harbor the N-terminal FG-repeat motif allowing for biomolecular condensates to form through LLPS around target genes and thereby deregulating their expression. NUP98’s capacity for LLPS has been shown to be evolutionarily conserved across metazoans, raising the possibility that therapy resistance may arise, in part, through transport-independent genome maintenance functions of nucleoporins. However, NUP98-mediated LLPS was established as protective in fruit flies, and thus, more broadly defining when NPC-proximal repair is protective versus when it increases toxic genome rearrangements may reveal therapeutically exploitable liabilities in treatment-resistant tumors. For example, dsbNET-mediated juxtaposition of lesions in PARP inhibitor-treated BRCA1-deficient cells has raised the possibility that inappropriate nuclear-envelope capture could, in some contexts, promote harmful rearrangements rather than genome preservation [31].

## Concluding remarks

The NPC has long been appreciated as the gatekeeper of nuclear transport. Together, these studies support a broader view in which NPC-associated environments and off-pore nucleoporin pools help cells manage DNA lesions that are unusually dangerous to repair in place. Across systems, three themes recur: the spatial quarantine of hazardous lesions, the shaping of repair outcome within specialized perinuclear environments, and the local containment of transcription-associated damage by mobile nucleoporins acting on chromatin.

This framework does not imply that all nucleoporin-dependent repair is direct, pore-anchored, or fully transport-independent. On the contrary, the field is marked by important evidentiary boundaries. Yet even with these limitations, a coherent picture is emerging. Nucleoporins do not simply accompany genome maintenance; under selected conditions, they help organize where repair occurs, when recombination is allowed, and how cells balance fidelity against survival. This view of the NPC as a spatial organizer of high-risk DNA lesions helps integrate diverse findings from yeast, flies, and mammals while clarifying the mechanistic questions that remain.
